# Outcomes at five to eight years of age for children with Hirschsprung’s disease

**DOI:** 10.1136/archdischild-2020-320310

**Published:** 2020-11-02

**Authors:** Benjamin Saul Raywood Allin, Charles Opondo, Timothy John Bradnock, Simon Edward Kenny, Jennifer J Kurinczuk, Gregor M Walker, Marian Knight, NETS collaboration, Mohammad Ahmad

**Affiliations:** 1 National Perinatal Epidemiology Unit, University of Oxford, Oxford, Oxfordshire, UK; 2 Paediatric Surgery, Royal Hospital for Children, Glasgow, UK; 3 Alder Hey Children's Hospital NHS Foundation Trust, Liverpool, UK

**Keywords:** gastroenterology, epidemiology

## Abstract

**Objective:**

This study describes core outcomes of Hirschsprung’s disease (HD) in a UK-wide cohort of primary school-aged children.

**Design:**

A prospective cohort study conducted from 1 October 2010 to 30 September 2012. Outcomes data were collected from parents and clinicians when children were 5–8 years of age, and combined with data collected at birth, and 28 days and 1 year post diagnosis.

**Setting:**

All 28 UK and Irish paediatric surgical centres.

**Participants:**

Children with histologically proven HD diagnosed at <6 months of age.

**Main outcome measures:**

NETS^1HD^ core outcomes.

**Results:**

Data were returned for 239 (78%) of 305 children. Twelve children (5%) died prior to 5 years of age.

Of the 227 surviving children, 30 (13%) had a stoma and 21 (9%) were incontinent of urine. Of the 197 children without a stoma, 155 (79%) maintained bowel movements without enemas/washouts, while 124 (63%) reported faecal incontinence. Of the 214 surviving children who had undergone a pull-through operation, 95 (44%) underwent ≥1 unplanned reoperation. 89 unplanned reoperations (27%) were major/complex.

Of the 83 children with returned PedsQL scores, 37 (49%) had quality of life scores, and 31 (42%) had psychological well-being scores, that were ≥1 SD lower than the reference population mean for children without HD.

**Conclusion:**

This study gives a realistic picture of population outcomes of HD in primary school-aged children in the UK/Ireland. The high rates of faecal incontinence, unplanned procedures and low quality of life scores are sobering. Ensuring clinicians address the bladder, bowel and psychological problems experienced by children should be a priority.

**What is already known on this topic?**

- There have been no previous population-based studies describing outcomes of Hirschsprung’s disease (HD) in primary school-aged children.
- The majority of existing studies are small, single institution, retrospective cohort studies, and therefore do not allow accurate conclusions to be drawn regarding the overall health and well-being of children with HD.
- A core outcome set was developed in 2017, identifying the 10 outcomes that are deemed most important by key stakeholders in defining successful treatment of children with HD.

**What this study adds?**

- The majority of children with HD are likely to have continence issues, and highly likely to have undergone multiple unplanned reoperations.
- The study provides a realistic picture of primary-school aged outcomes, which can be used for the counselling of parents.
- The study should also prompt health professionals to reflect on how children with HD are managed, the multidisciplinary team is used and services are configured.

## Background and objectives

By conducting interviews with 44 parents of children who underwent neonatal surgery, including parents of children with Hirschsprung’s disease (HD), Hinton *et al* 1 identified six aspects of care that affected a parent’s experience. Central to this experience was the ability of surgeons to help parents understand their child’s diagnosis and the journey ahead. In general, feedback from the parents interviewed was that the hospitals were not able to supply them with sufficient information to address their concerns. One way to improve the experience of parents of children who undergo surgery for HD is to increase the amount of reliable, easily understandable information that hospitals are able to give them regarding their child’s diagnosis and likely long term health.^1^

At present however, with the exception of data from a few groups,^2–5^ there is little information available on which to base counselling of parents. It is therefore very difficult for surgeons to reference reliable information to help parents understand their child’s diagnosis and probable treatment.

In order to produce information that could improve the counselling of parents of children with HD, the overall aim of this study was to collect data from clinicians and parents, and use it to describe core outcomes^6^ at primary school age for a British and Irish cohort of children with HD.

## Methods

### Study design and setting

Between 2010 and 2012, a prospective population-based cohort study (The British Association of Paediatric Surgeons Congenital Anomalies Surveillance System HD Study) was conducted, collecting data relating to the presentation, early management and early outcomes of children diagnosed with HD in all 28 paediatric surgical centres in the UK and Ireland.^2^ Children from 20 of these sites were subsequently followed up at 5–8 years of age. The follow-up comprised two components, one collecting data from parents, and one collecting data from clinicians. Data from both sources were combined with the previously collected data, and the amalgamated dataset used to describe core outcomes at 5–8 years of age for this cohort.

### Participants

The original cohort data were collected for any live-born infant who was diagnosed with HD between the 1 October 2010 and 31 September 2012 in one of the 28 paediatric surgical centres in the UK and Ireland, and who was <6 months of age at the time of diagnosis. HD was defined as an absence of ganglia in the enteric nervous system of the distal bowel (aganglionosis). Infants diagnosed after 6 months of age were excluded. This cohort consisted of 305 infants.

### Outcomes

In 2017, the NETS^1HD^ core outcome set was developed by over 100 paediatric surgeons, paediatric gastroenterologists, specialist nurses, parents of children with HD, adults who had previously been treated for HD and other key stakeholders.^6^ This core outcome set identified the outcomes most important in defining successful treatment of a child with HD. The outcomes reported in this study are those defined by the NETS^1HD^ core outcome set (box 1).

Box 1 **Outcome definition as per the NETS^1HD^ core outcome set**

1. *Death* with cause classified as due to:
  - A complication of treatment (excluding Hirschsprung’s-associated enterocolitis)
  - Hirschsprung’s-associated enterocolitis.
  - An associated anomaly.
  - Other condition.
2. *Unplanned reoperation* with the indication for re-operation specified. Unplanned was defined as any procedure not considered part of routine postintervention practice. This therefore excluded stoma closures performed separately as part of a planned three-stage pull-through procedure, but included all anal dilatations/calibrations performed under anaesthesia, as opinion varies as to whether these are to be considered part of routine care or not. Re-operation included all procedures performed as a direct result of the diagnosis or treatment of the child’s Hirschsprung’s disease (HD), *and* all episodes of general anaesthesia that were required as a direct result of the diagnosis or treatment of the child’s HD, regardless of whether an operative intervention was undertaken, for example, examinations under anaesthesia. As the outcome of interest was *re*-operation, not operation, only those operations occurring after the child’s pull-through were included. Where multiple procedures were undertaken at the same time, these were counted as one additional unplanned reoperation, and classified as the most significant procedure performed. Indications for the operations were classified according to National Institute for Health and Care Excellence (NICE) criteria as minor (eg, botox injection, abscess drainage), intermediate (eg, antegrade continence enema formation, incisional hernia repair) or major/complex (eg, intestinal resections, stoma formation) (NICE Guideline NG45—routine preoperative tests for elective surgery).
3. *Faecal incontinence*, defined as involuntary passage of faecal matter in an inappropriate place, and with the severity graded as:
  - Occasionally (eg, once to twice per week) either with or without causing social problems;
  - Every day but without social problems;
  - Constant and causing social problems.
4. *Urinary incontinence*, defined as involuntary voiding of urine that was constant, associated with social problems, or requiring catheterisation.
5. *Permanent stoma* as a direct result of the diagnosis or treatment of the child’s HD, including where the decision for a stoma had been made out of child or parental preference, or for continence management. Permanent stoma was defined as one that was created without the intention of later reversal. Indication for stoma formation was reported.
6. *Hirschsprung’s-associated enterocolitis*, clinician decision to admit and treat for Hirschsprung’s-associated enterocolitis.
7. *Objective score of bowel function*, as measured by the Paediatric Incontinence and Constipation Score.^8^
8. *Voluntary bowel movements without need for enemas or rectal or colonic irrigation*.
9. *Quality of life*, as measured by the total scale score for the parent proxy-reported PedsQL questionnaire for children aged 5–7 years.
10. *Psychological stress*, as measured by the psychosocial health summary score for the parent proxy-reported PedsQL questionnaire for children aged 5–7 years.

### Data sources and subpopulations for outcome reporting

Data were collected from four sources, a 28-day clinician form, a 1 year clinician form, a 5–8 years parent form and a 5–8 years clinician form. Not all children whose data were used in the analyses had data returned for all four forms, and the numbers of children on which each analysis was conducted therefore varied (box 2).

Box 2 **Data sources and populations for outcome reporting**

**Mortality**

Five-year mortality could be determined from both 5–8 years data collection forms and is therefore described in the cohort of 239 children who had either form returned. Minimum and maximum estimates of mortality are also described for the total cohort of 305 children.

**Bowel and bladder function**

Data relating to faecal incontinence, urinary incontinence, Hirschsprung’s-associated enterocolitis, presence of a permanent stoma and voluntary bowel movements without need for enemas or rectal or colonic washouts, were collected on both the 5–8 years clinician data collection form and the 5–8 years parent data collection form. The first four of these outcomes are therefore described in the population of 227 children who were alive at 5 years of age and had either 5–8 years data collection form returned. Voluntary bowel movements is described in the population of 197 children who were alive at 5 years of age, had either 5–8 years data collection form returned and who did not have a stoma at the time of completion of the data collection form.

**Number of unplanned reoperations**

Describing the number of unplanned reoperations, a child underwent in the first 5–8 years of life required return of data from the 5–8 years clinician data collection form. Therefore, numbers of unplanned reoperations are described in the cohort of 214 children who had survived to 5 years of age, had undergone a pull-through procedure and had a 5–8 years clinician form returned.

**Quality of life and Paediatric Incontinence and Constipation Score (PICS)**

Quality of life, psychological stress and the PICS data were only collected using the 5–8 years parent form. Quality of life and psychological stress are therefore described in the population of 83 children who had a 5–8 years parent data collection form returned and the PICS is described in the 72 children who had a 5–8 years data collection form returned and who did not have a stoma at time of completion of the form.

### Statistical analyses

Counts and proportions, means and 95% CIs and medians and IQRs, were used as appropriate to describe the 10 outcomes of interest. Outcomes were described in the cohort as a whole and in two subgroups of infants, those with short segment HD and those with either long-segment or total colonic HD. Counts and proportions of children who had undergone none, one, two, three and four or more unplanned reoperations between the time of their pull-through procedure, and 5–8 years of age were described. A PedsQL total scale score, and a PedsQL psychosocial health summary score were calculated for each infant as per the scale developer’s instructions. The mean (SD) total scale score and psychosocial health summary score were calculated, and compared using an unpaired t-test with the equivalent scores in an established reference population.^7^ No appropriately sized British reference population was available for comparison against, and therefore a reference population from a similar, high-income Western country was sought. The identified reference population was developed in a cohort of American children, and is the largest currently available. The number and proportion of infants with health-related quality of life, and psychosocial health related quality of life scores that were clinically significantly lower than children unaffected by HD were also calculated. A clinically significant reduction in either quality of life score was defined, as per the scale developer’s instructions, as more than 1 SD below the mean for the appropriate scale in the defined reference population.^7^ Medians and IQRs for the incontinence and constipation scores derived from the PICS were described. The counts and proportions of infants with constipation or impaired continence, as evidenced by a score of less than the age-specific lower 95% confidence limit on the appropriate score were also described. For children aged 5–8 years, these lower 95% CI levels were 23.2 on the incontinence score and 20.1 on the constipation score.^8^

## Results

### Follow-up

Of the 28 paediatric surgical centres in the UK and Ireland, 8 did not participate in the 5–8 years follow-up due to extensive delays in permissions processes. Fifty-three infants (17% of the original cohort) were treated in these eight centres. Comparison of demographics, initial management and outcomes at 1 year post-diagnosis, between infants treated in participating and non-participating sites are described in online supplemental tables 1 and 2.

10.1136/archdischild-2020-320310.supp1 Supplementary data

**Figure 1:**
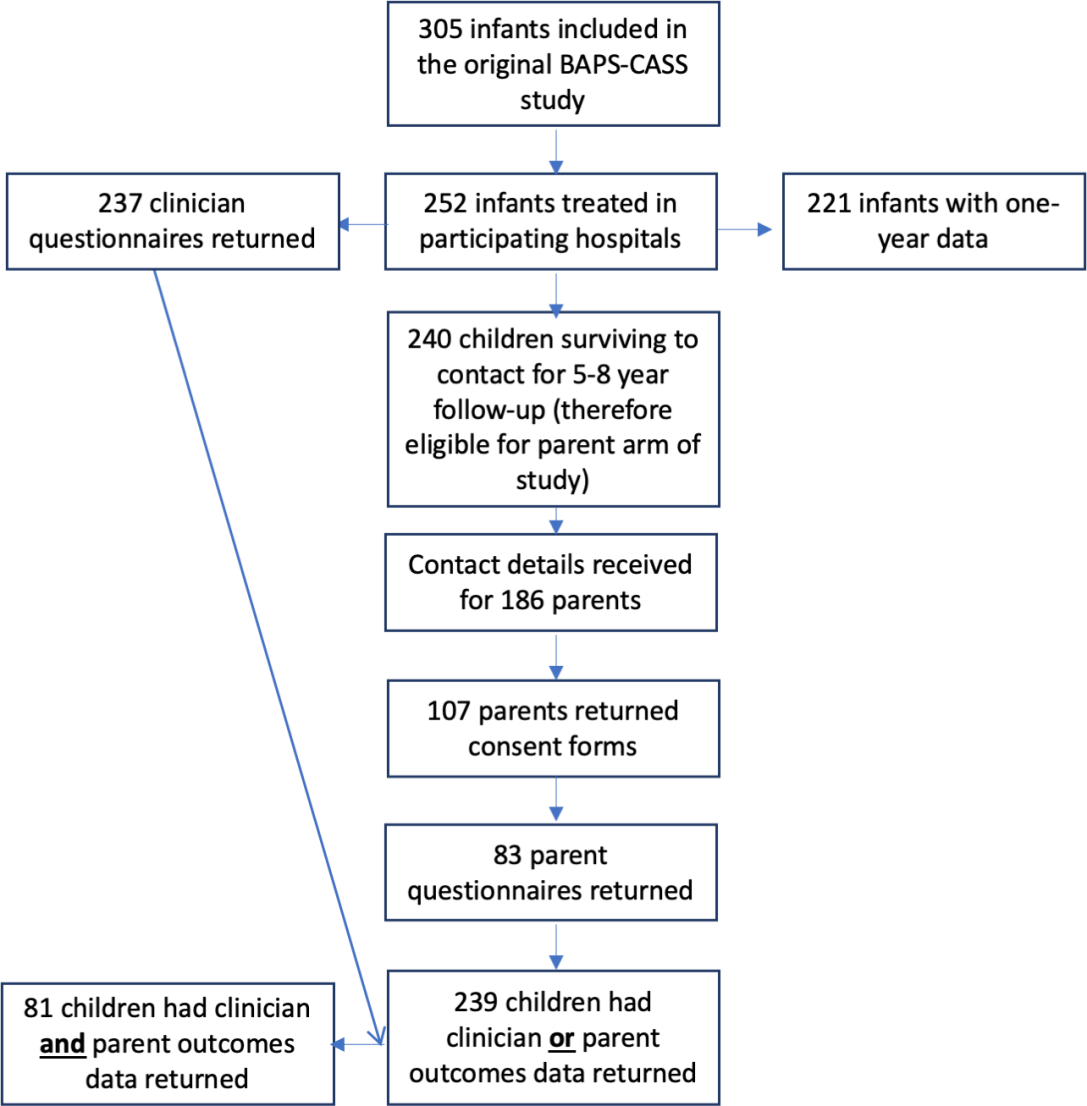
Data return for the NETS^2HD^ study. BAPS-CASS, British Association of Paediatric Surgeons Congenital Anomalies Surveillance System.

**Figure 2:**
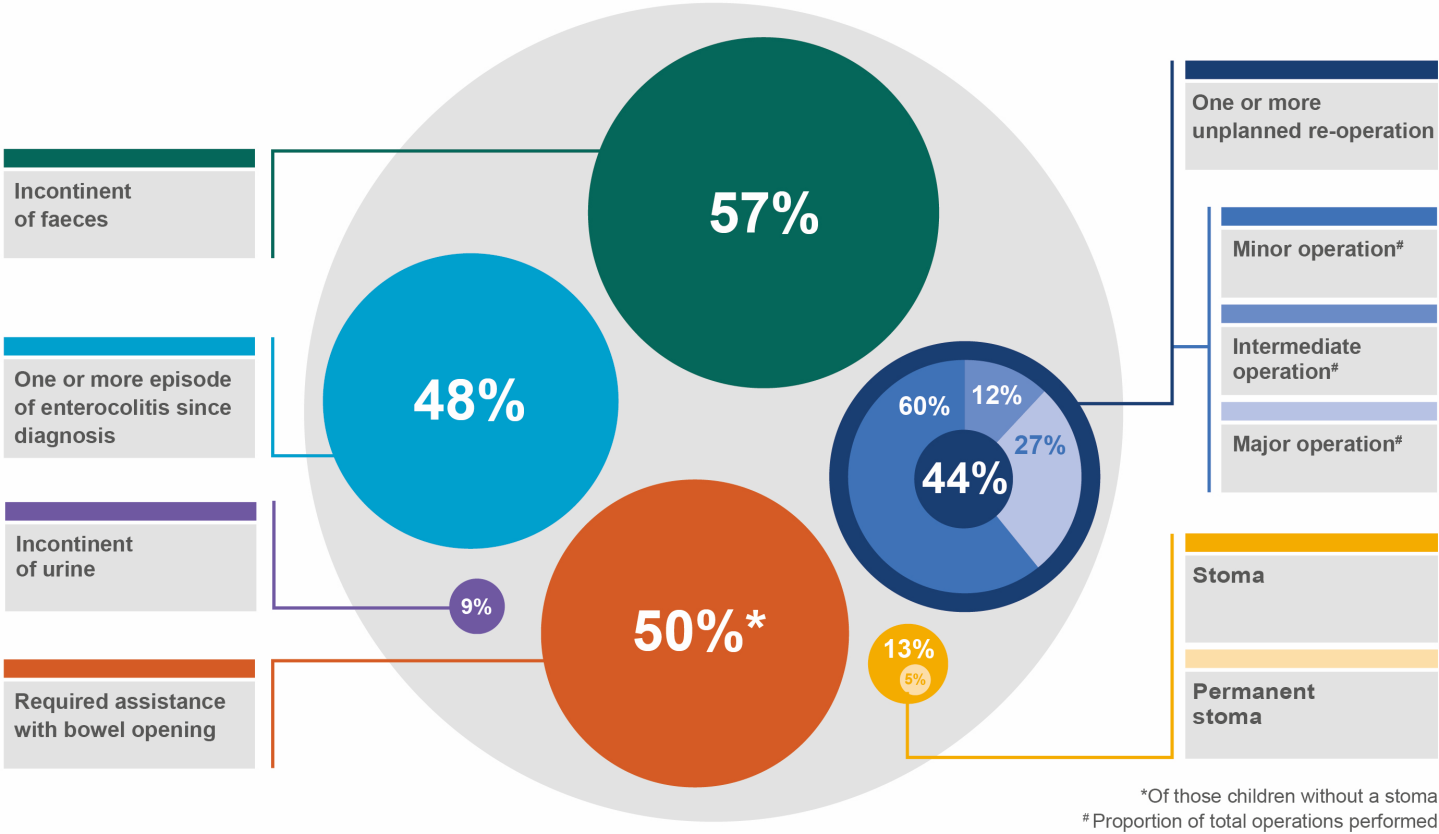
Proportion of children reporting key outcomes at 5–8 years of age.

Of the 252 children (83% of the original cohort) diagnosed in sites participating in the 5–8 years follow-up, data were received for 239 (95%). For the 227 children from participating centres that had outcomes data returned, and who survived to follow-up, the median (IQR) age at completion of the data collection form was 6.8 years (6.2–7.3 years). The youngest child who had a questionnaire completed was aged 5 years and 6 months, and the oldest was 8 years and 2 months. The age at diagnosis was earlier in children lost to follow-up than those who were followed up. There were no other differences between children with and without either form of follow-up, while those with parental follow-up were more likely to have been treated in a low volume centre (<median number of cases treated/year), and more likely to have been treated using a laparoscopic pull-through than those without parental follow-up (online supplemental tables 3-5). Figure 1 describes where loss to follow-up occurred, and figure 2 summarises overall outcomes for the cohort.

### Mortality

Eight infants (3%) died prior to 1 year of age and a further four infants (2%) died between 1 and 5 years of age, giving an overall case-fatality for those children with complete follow-up of 5% (95% CI 3% to 9%) (table 1). Based on the proportion of children who were known to have died between 1 and 5 years of age, it would be anticipated that one of the 66 children who were lost to follow-up would also have died between 1 and 5 years of age. As only one additional child was anticipated to have died in the proportion of children who were lost to follow-up, the maximum and minimum estimates of the 5-year case fatality for the entire cohort of 305 children were the same (4%, 95% CI 2% to 7%).

**Table 1:** Causes of death

	n (%)*
Death prior to 5 years of age	**N=239**
No	227 (95%)
Yes	12 (5%)
Cause of death	**N=12**
Direct consequence of HD or its treatment	2 (17%)
Hirschsprung’s-associated enterocolitis	0 (0%)
Associated anomaly	7 (58%)
Other causes	3 (25%)

*Percentage of children with returned data.

HD, Hirschsprung’s disease.

### Bowel and bladder function outcomes

Of the 227 children who were known to have survived to 5 years of age, 30 (13%) had a stoma, 11 of which (37%) were formed without the intention of later reversal (table 2). Two (18%) of the 11 permanent stomas were formed because the child had total intestinal aganglionosis, 2 (18%) were formed in children with developmental delay in whom the pull-through had been unsuccessful, 5 (45%) were formed in preference to performing a pull-through and 2 (18%) were formed to aid continence management after a previous pull-through. The five children for whom a stoma was formed in preference to a pull-through were considered not suitable for a pull-through either because of significant neuro-disability or additional comorbidities.

**Table 2:** Bowel and bladder function

	n (%)*
	**N=227 children**
Any stoma	
No	196 (87%)
Yes	30 (13%)
Permanent stoma	
No	215 (95%)
Yes	11 (5%)
Faecal incontinence	
No	97 (43%)
Yes	129 (57%)
At least one episode of Hirschsprung’s-associated enterocolitis since diagnosis	
No	119 (52%)
Yes	108 (48%)
Number of episodes Hirschsprung’s-associated enterocolitis in past 12 months	
None	216 (95%)
One	8 (4%)
Two or more	3 (1%)
Urinary incontinence	
No	205 (91%)
Yes	21 (9%)
	**N=197† children**
Assistance required to maintain voluntary bowel movements	
None	99 (50%)
Laxatives	56 (28%)
Regular enemas	18 (9%)
Rectal irrigation	17 (9%)
Antegrade continence enema	5 (3%)
Unknown	2 (1%)

*Percentage of those with complete data.

†Described in those children without a stoma.

Of the 197 children without a stoma, 124 (63%) were incontinent of faeces. A further 5 (17%) of the 30 children with a stoma also passed mucus, or other matter, per rectum, accidentally, in inappropriate places, despite the presence of the stoma. Such accidental passage of matter per rectum in an inappropriate place met the definition used for faecal incontinence in the core outcome set, and therefore, overall, at 5–8 years of age, 129 children (57%) were classified as incontinent of faeces. The severity of faecal incontinence was reported as occurring once or twice per week for 51 children (40%), every day but without social problems for 36 children (28%), and constantly and causing social problems for 32 children (25%). The severity of faecal incontinence was unknown for 10 children (7%). In the cohort of 175 children with isolated HD, that is, those without an additional congenital anomaly or known syndrome, 105 (60%) were incontinent of faeces at 5–8 years of age. For the 72 children where faecal continence status was reported by both surgeons and parents, parents reported their child as incontinent in 65 instances (91%). Of these 65, surgeons only reported 35 (54%) as incontinent of faeces. There were no instances where surgeons reported children as incontinent, but parents did not.

All other outcomes are described in tables 2 and 3.

**Table 3:** Unplanned reoperations

	n (% of children)*
	N=214 children
Number of unplanned reoperations	
None	119 (56%)
One	36 (17%)
Two	18 (8%)
Three	11 (5%)
Four or more	30 (14%)
	n (% of unplanned reoperations)*
	**N=326 operations**
Type of unplanned reoperation	
Minor	197 (60%)
Intermediate	40 (12%)
Major/Complex	89 (27%)

*Percentage of those with complete data.

### Quality of life and bowel function scores

PedsQL total scale scores were calculable for 76 (92%) of the 83 children with a 5–8 years parent form returned. Psychosocial health summary scores were calculable for 74 (89%). The mean (SD) total scale score was 68.1 (20.62), and the mean psychosocial health summary score was 67.4 (20.96). These were statistically significantly lower (p<0.001) than the mean (SD) total scale score of 78.02 (16.44), and mean (SD) psychosocial health summary score of 81.24 (15.34) derived from the reference populations described by Varni *et al*.^7^ There were 37 children (49%) whose total scale score, and 31 (42%) whose psychosocial health summary score was more than 1 SD below the reference population means.^7^

Of the 72 children who had a 5–8 years parent form returned and who did not have a stoma at the time of completion of the form, 39 (54%) had sufficient PICS data returned to allow calculation of both the incontinence and constipation scores. The median (IQR) incontinence score for these children was 17.7 (14–23.5), and the median (IQR) constipation score was 16.2 (10.5–21.5). There were 29 children (74%) who met criteria for impaired continence, and 27 (69%) who met criteria for evidence of constipation.

### Outcomes according to length of affected bowel

Histological confirmation of the transition zone is only possible for children who have undergone definitive surgery, and therefore the outcomes described only relate to the 214 children who underwent a pull-through procedure, 159 (74%) of whom had rectosigmoid disease, 46 (22%) of whom had long-segment disease and 9 (4%) of whom had total colonic disease. Three children (2%) with rectosigmoid disease died after definitive surgery but prior to 5 years of age, one as a direct result of the diagnosis or treatment of their HD, and two as a result of other causes. No children with long-segment HD died after surgery and prior to 5 years of age. All other outcomes are described in tables 4 and 5.

**Table 4:** Clinical outcomes described according to length of affected bowel

	Short segment	Long segment or total colonic
	**N=156**	**N=55**
Unplanned reoperation	n(%)*	n(%)*
None	95 (62%)	22 (40%)
One	26 (17%)	10 (18%)
Two	12 (8%)	6 (11%)
Three	5 (3%)	6 (11%)
Four or more	16 (10%)	11 (20%)
Stoma		
No	140 (90%)	40 (87%)
Yes	15 (10%)	7 (13%)
Permanent stoma		
No	152 (98%)	54 (98%)
Yes	3 (2%)	1 (2%)
Faecal incontinence		
No	68 (44%)	18 (33%)
Yes	87 (56%)	37 (67%)
At least one episode of Hirschsprung’s-associated enterocolitis		
No	83 (53%)	27 (49%)
Yes	73 (47%)	28 (51%)
Urinary incontinence		
No	142 (92%)	51 (93%)
Yes	13 (8%)	4 (7%)
Voluntary bowel movements without enemas or rectal or colonic washouts	**N=141†**	**N=48†**
Yes	113 (81%)	35 (73%)
No	26 (19%)	13 (27%)

*Percentage of those with complete data.

†Described in those children without a stoma.

**Table 5:** Quality of life and bowel function described according to length of affected bowel

	Short segment	Long segment or total colonic
	N=52*	N=22*
Quality of life		
Quality of life score ≥1 SD lower than reference population mean, n (%)*		
No	23 (50%)	12 (57%)
Yes	23 (50%)	9 (43%)
PedsQL total scale score, mean (SD)	69.4 (19.5)	67.8 (24.0)
Psychological stress		
Psychosocial health quality of life score ≥1 SD lower than reference population mean, n (%)*		
No	26 (59%)	12 (57%)
Yes	18 (41%)	9 (43%)
Psychosocial health summary score, mean (SD)	69.2 (19.3)	66.2 (23.2)
	N=47*†	N=20*†
PICS		
Normal continence, n (%)*	8 (30%)	1 (13%)
Impaired continence, n (%)*	19 (70%)	7 (88%)
Incontinence score, median (IQR)	19.5 (11–23.5)	20.5 (18–22.25)
No constipation, n (%)*	8 (30%)	4 (50%)
Constipation, n (%)*	19 (70%)	4 (50%)
Constipation score, median (IQR)	16 (11–21)	20.3 (10.3–23.3)

*Percentage of those with returned data.

†Described in those children without a stoma and with complete PICS data for both constipation and continence scores.

PICS, Paediatric Incontinence and Constipation Score.

## Discussion

The key finding from this study is that, during their primary school years, the health and well-being of children with HD treated in the UK and Ireland is significantly impaired. Approximately 1 in 5 children in this population-wide cohort required enemas or rectal or colonic washouts to maintain voluntary bowel movements, 1 in 10 were incontinent of urine, approximately three-fifths were incontinent of faeces and nearly half underwent at least one unplanned reoperation, a quarter of which were classified as major or complex. For over half of those children who were incontinent of faeces, soiling occurred on a daily basis, and in a quarter, was resulting in significant social problems. Even when children with long-segment and total colonic HD, or those with a syndrome or additional congenital anomaly were excluded, continence for the remaining children remained poor compared with unaffected peers, for whom the prevalence of faecal incontinence and urinary incontinence are estimated at 2%–4%, and 3%, respectively.^9 10^ With the quality of life scores reported here on a par with those of children with end-stage renal disease and cancer, the overall impact of HD on a child’s life appears significant.^11^

While one of the key strengths of this study was the inclusion of parent-reported outcomes data, facilitating inclusion of these data has undoubtedly also resulted in one of the key limitations, the lower than anticipated data return rate. In participating sites, the protracted approvals process resulted in the first parental questionnaire being sent out over a year after the originally planned study start date, thereby significantly reducing the time available for data collection. Despite these difficulties, 78% of the originally identified cohort had either clinician or parent data returned, and 27% had data returned from both sources. As the reasons for non-return of parent-reported outcomes data are many and varied, it is difficult to predict what impact this loss to follow-up may have had on the outcomes described. However, as there were no meaningful differences in characteristics, management or 1-year outcomes between children with and without follow-up, we do not believe that this loss to follow-up has significantly affected the representativeness of the results.

In older children and young adults with HD, the proportion who are incontinent of faeces has been reported as ranging from 20% to 50%,^4 12–14^ urinary incontinence has been reported to affect approximately 2%^15^ and quality of life and mental health have been suggested to be little different from people without HD.^3 16–18^ Outcomes for the cohort of children studied here therefore appear to be worse than for those reported elsewhere. It is unclear why this is the case, but there are two plausible explanations. First, including parent-reported outcomes data may have increased the proportion of adverse outcomes that are reported, and second, outcomes may vary over time, and the age at which outcomes were reported in this work represents a point where they are at their worst. Evidence exists to support both of these theories.^17–22^

Overall, this study gives a realistic picture of the population outcomes for primary school-aged children with HD in the UK and Ireland. The high rates of faecal incontinence, need for enemas or colonic washouts, unplanned reoperations and low quality of life scores are sobering, and give pause to reflect on the way children with HD are currently managed. These results, when placed in the context of probable improvement with age,^17 18 21–23^ can provide a useful data source for counselling of parents of children with HD. Regardless of any later improvement in outcomes, early childhood is likely to be a tumultuous time for families of children with HD, and therefore, ensuring regular follow-up, with easy access to the multidisciplinary team, bowel and bladder management programmes and psychological support should be a priority. We would strongly advocate for access to such services becoming the norm across all centres providing surgery for children with HD in the UK and Ireland.

## Data Availability

All data relevant to the study are included in the article or uploaded as supplementary information.
