# Supplementary material for: Outcomes at five to eight years of age for children with Hirschsprung’s disease
Source: Arch Dis Child. 2020 Nov 2;106(5):484–90. doi: 10.1136/archdischild-2020-320310 (PMC8070639; doi:10.1136/archdischild-2020-320310)
Supplement: Supplementary data [file archdischild-2020-320310supp001.pdf]

S1: Characteristics and management of infants treated in participating and non-participating sites

	**Non-participating sites**	**Participating sites**	
	**N=53**	**N=252**	
	**n(%)***	**n(%)***	**p-value**
**Diagnosed in a low volume centre (< national median number of cases/year)**			<0·001
No	0 (0·0%)	141 (56·0%)	
Yes	53 (100·0%)	111 (44·0%)	
**Ethnicity**			0·68
White	44 (84·6%)	216 (86·7%)	
BME	8 (15·4%)	33 (13·3%)	
**Gestational age at birth**			0·10
Term	49 (94·2%)	215 (86·0%)	
Pre-term	3 (5·8%)	35 (14·0%)	
**Birthweight**			0·060
2500g or more	49 (96·1%)	211 (86·8%)	
Less than 2500g	2 (3·9%)	32 (13·2%)	
**Sex**			0·46
Male	38 (73·1%)	196 (77·8%)	
Female	14 (26·9%)	56 (22·2%)	
**Family history of HD**			<0·001
No	41 (78·8%)	234 (94·0%)	
Yes	11 (21·2%)	15 (6·0%)	
**Associated anomaly**			0·14
Isolated HD	47 (88·7%)	184 (73·6%)	
Down's syndrome	2 (3.8%)	26 (10%)	
Other identified syndrome	2 (3.8%)	21 (8%)	
Isolated additional anomaly	2 (3·8%)	19 (7·6%)	
**Age at presentation**			0·94
1-7 days	47 (88·7%)	219 (86·9%)	
8-28 days	2 (3·8%)	11 (4·4%)	
More than 28 days	4 (7·5%)	22 (8·7%)	

* Percentage of those with complete data

Allin BSR, *et al. Arch Dis Child* 2021; 106:484–490. doi: 10.1136/archdischild-2020-320310

S1: Characteristics and management of infants treated in participating and non-participating sites - Continued

	Non-participating sites	Participating sites	
	N=53	N=252	
	n(%)*	n(%)*	p-value
**Age at diagnosis**			0·82
<31 days	47 (88·7%)	213 (85·2%)	
31-60 days	2 (3·8%)	10 (4·0%)	
61-90 days	3 (5·7%)	13 (5·2%)	
91-120 days	1 (1·9%)	4 (1·6%)	
121-150 days	0 (0·0%)	2 (0·8%)	
>150 days	0 (0·0%)	8 (3·2%)	
**Transition zone (as defined at surgery)**			0·35
Rectosigmoid	31 (67·4%)	168 (74·3%)	
Long segment (proximal to sigmoid colon)	11 (23·9%)	49 (21·7%)	
Total colonic aganglionosis	4 (8·7%)	9 (4·0%)	
**Stoma created prior to pull-through**			0·18
No	38 (71·7%)	156 (61·9%)	
Yes	15 (28·3%)	96 (38·1%)	
**Definitive surgery within a year of diagnosis**			0·95
No	4 (7·7%)	17 (7%)	
Yes	48 (92·3%)	225 (93%)	
**Staged definitive procedure**			0·14
No	38 (77·6%)	153 (66·8%)	
Yes	11 (22·4%)	76 (33·2%)	
**First approach to the pull-through**			0·034
Definitive surgery not performed prior to one year	5 (9·6%)	17 (7%)	
Open	17 (32·7%)	87 (36%)	
Laparoscopic	9 (17·3%)	42 (17·4%)	
Trans-anal	3 (5·8%)	50 (20·7%)	
Combined laparoscopic and trans-anal	18 (34·6%)	46 (19·0%)	
**Rectal dissection technique**			0·007
Submucosal (Soave)	29 (60·4%)	119 (52%)	
Peri-rectal (Swenson)	9 (18·8%)	17 (7·4%)	
Posterior (Duhamel)	10 (20·8%)	93 (40·6%)	
	**Median (IQR)**	**Median (IQR)**	
**Weight at stoma formation**			0·23
Grams	3800 (3340, 4200)	3500 (3000, 4000)	
**Weight at definitive surgery**			0·052
Kilograms	6·12 (5·2, 6·92)	5·43 (4·21, 6·55)	

* Percentage of those with complete data

Allin BSR, *et al. Arch Dis Child* 2021; 106:484–490. doi: 10.1136/archdischild-2020-320310

S2: One year outcomes for infants who underwent definitive surgery in participating and non-participating sites

	Non-participating site	Participating site	
	N=36	N=204	
	n(%)*	n(%)*	p-value
**Revision surgery within a year of diagnosis**			0·42
No	30 (100·0%)	187 (97·9%)	
Yes	0 (0·0%)	4 (2·1%)	
**Post pull-through stoma within a year of diagnosis**			0·11
No	25 (80·6%)	160 (90·4%)	
Yes	6 (19·4%)	17 (9·6%)	
**Post-operative HAE suspected**			0·70
No	25 (71·4%)	137 (68·2%)	
Yes	10 (28·6%)	64 (31·8%)	
**One or more unplanned reoperations within a year of diagnosis**			0·72
No	31 (88·6%)	165 (85·9%)	
Yes	4 (11·4%)	27 (14·1%)	
**Total number of unplanned operations within a year of diagnosis**			0·81
None	31 (88·6%)	165 (85·9%)	
One	3 (8·6%)	15 (7·8%)	
Two	1 (2·9%)	8 (4·2%)	
Three	0 (0·0%)	4 (2·1%)	

*Percentage of those with complete data

Allin BSR, *et al. Arch Dis Child* 2021; 106:484–490. doi: 10.1136/archdischild-2020-320310

S3: Characteristics and management of infants treated in participating sites with and without five to eight year data

	No data returned	Data returned	
	N=13	N=239	
	n(%)*	n(%)*	p-value
**Diagnosed in a low volume (< median) centre**			0·46
No	6 (46·2%)	135 (56·5%)	
Yes	7 (53·8%)	104 (43·5%)	
**Ethnicity**			0·28
White	10 (76·9%)	206 (87·3%)	
BME	3 (23·1%)	30 (12·7%)	
**Gestational age at birth**			0·26
Term	9 (75·0%)	206 (86·6%)	
Pre-term	3 (25·0%)	32 (13·4%)	
**Birthweight**			0·21
2500g or more	9 (75·0%)	202 (87·4%)	
Less than 2500g	3 (25·0%)	29 (12·6%)	
**Sex**			0·54
Male	11 (84·6%)	185 (77·4%)	
Female	2 (15·4%)	54 (22·6%)	
**Family history of HD**			0·35
No	13 (100·0%)	221 (93·6%)	
Yes	0 (0·0%)	15 (6·4%)	
**Associated anomaly**			0·39
Isolated HD	7 (58·3%)	177 (74·4%)	
Down’s Syndrome	3 (23%)	23 (10%)	
Other identified syndrome	1 (8%)	20 (8%)	
Isolated additional anomaly	1 (8·3%)	18 (7·6%)	
**Age at presentation (days)**			0·55
1-7 days	10 (76·9%)	209 (87·4%)	
8-28 days	1 (7·7%)	10 (4·2%)	
More than 28 days	2 (15·4%)	20 (8·4%)	
**Age at diagnosis**			0·022
<31 days	9 (69·2%)	204 (86·1%)	
31-60 days	0 (0·0%)	10 (4·2%)	
61-90 days	3 (23·1%)	10 (4·2%)	
91-120 days	1 (7·7%)	3 (1·3%)	
121-150 days	0 (0·0%)	2 (0·8%)	
>150 days	0 (0·0%)	8 (3·4%)	
**Transition zone (as defined at surgery)**			0·75
Rectosigmoid	9 (75·0%)	159 (74·3%)	
Long segment (proximal to sigmoid colon)	3 (25·0%)	46 (21·5%)	
Total colonic aganglionosis	0 (0·0%)	9 (4·2%)	

* Percentage of those with complete data
BME – Black or other minority ethnicity

S3: Characteristics and management of infants treated in participating sites with and without five to eight year data - Continued

	No data returned	Data returned	
	N=13	N=239	
	n(%)*	n(%)*	p-value
**Stoma created prior to pull-through**			0·54
No	7 (53·8%)	149 (62·3%)	
Yes	6 (46·2%)	90 (37·7%)	
**Definitive surgery (including permanent stoma) confirmed as occurring within one year of diagnosis**			0·31
No	0 (0·0%)	17 (7·4%)	
Yes	12 (100·0%)	213 (92·6%)	
**Staged definitive procedure**			0·52
No	7 (58·3%)	146 (67·3%)	
Yes	5 (41·7%)	71 (32·7%)	
**First approach to the pull-through**			0·56
Pull-through procedure not performed within one year of diagnosis	0 (0·0%)	17 (7·4%)	
Open	6 (50·0%)	81 (35·2%)	
Laparoscopic	3 (25·0%)	39 (17·0%)	
Trans-anal	2 (16·7%)	48 (20·9%)	
Combined laparoscopic and trans-anal	1 (8·3%)	45 (19·6%)	
**Rectal dissection technique**			0·32
Submucosal (Soave)	5 (41·7%)	114 (52·5%)	
Peri-rectal (Swenson)	0 (0·0%)	17 (7·8%)	
Posterior (Duhamel)	7 (58·3%)	86 (39·6%)	
	**Median (IQR)**	**Median (IQR)**	
**Weight at stoma formation**			0·21
Grams	4100 (3500, 5000)	3475 (3000, 4000)	
**Weight at definitive surgery**			0·99
Kilograms	5·5 (4·5, 7·32)	5·4 (4·2, 6·55)	

*Percentage of those with complete data

Allin BSR, *et al. Arch Dis Child* 2021; 106:484–490. doi: 10.1136/archdischild-2020-320310

S4: Outcomes at one year post diagnosis for children with and without five to eight year data returned

Based upon those children who had undergone definitive surgery in a participating hospital, and for whom one-year outcomes data had been returned.

	No data returned	Data returned	
	N=11[#]	N=193[#]	
	n(%)*	n(%)*	p-value
**Revision surgery within a year of diagnosis**			0·63
No	10 (100·0%)	177 (97·8%)	
Yes	0 (0·0%)	4 (2·2%)	
**Post pull-through stoma within a year of diagnosis**			0·78
No	7 (87·5%)	153 (90·5%)	
Yes	1 (12·5%)	16 (9·5%)	
**Post-operative HAE suspected**			0·32
No	9 (81·8%)	128 (67·4%)	
Yes	2 (18·2%)	62 (32·6%)	
**One or more unplanned reoperation within a year of diagnosis**			0·65
No	9 (81·8%)	156 (86·2%)	
Yes	2 (18·2%)	25 (13·8%)	

[#] Those children who had undergone definitive surgery prior to one year of age, and for whom one-year outcomes data were returned

* Percentage of those with complete data

Allin BSR, *et al. Arch Dis Child* 2021; 106:484–490. doi: 10.1136/archdischild-2020-320310

S5: Characteristics and management of infants with and without parental follow-up data

	No Parental Data N=157 n(%)*	Parental Data N=83 n(%)*	p-value
Low volume centre (<median number of cases/year)			0.006
No	97 (61.8%)	36 (43.4%)	
Yes	60 (38.2%)	47 (56.6%)	
Ethnicity			0.22
White	131 (84.5%)	74 (90.2%)	
Non-white	24 (15.5%)	8 (9.8%)	
Gestational age at birth			0.89
Term	136 (87.2%)	72 (87.8%)	
Pre-term	20 (12.8%)	10 (12.2%)	
Birthweight			0.77
2500g or more	132 (87.4%)	71 (88.8%)	
Less than 2500g	19 (12.6%)	9 (11.2%)	
Sex			0.65
Male	125 (79.6%)	64 (77.1%)	
Female	32 (20.4%)	19 (22.9%)	
Family history of Hirschsprung's disease			0.21
No	148 (95.5%)	75 (91.5%)	
Yes	7 (4.5%)	7 (8.5%)	
Associated anomaly			0.97
Isolated Hirschsprung's disease	120 (76.9%)	62 (75.6%)	
Syndromic	25 (16.0%)	14 (17.1%)	
Isolated additional anomaly	11 (7.1%)	6 (7.3%)	
Age at presentation (days)			0.23
1-7 days	139 (88.5%)	70 (84.3%)	
8-28 days	4 (2.5%)	6 (7.2%)	
More than 28 days	14 (8.9%)	7 (8.4%)	
Age at diagnosis			0.86
<31 days	131 (84.0%)	72 (86.7%)	
31-60 days	5 (3.2%)	4 (4.8%)	
61-90 days	10 (6.4%)	3 (3.6%)	
91-120 days	3 (1.9%)	1 (1.2%)	
121-150 days	1 (0.6%)	1 (1.2%)	
>150 days	6 (3.8%)	2 (2.4%)	
Transition zone (as defined at surgery)			0.64
Rectosigmoid	113 (75.8%)	52 (70.3%)	
Long segment (proximal to sigmoid colon)	30 (20.1%)	19 (25.7%)	
Total colonic aganglionosis	6 (4.0%)	3 (4.1%)	
Stoma created prior to pull-through			0.32
No	103 (65.6%)	49 (59.0%)	
Yes	54 (34.4%)	34 (41.0%)	
Definitive surgery confirmed as occurring prior to one year			0.27
No	9 (5.8%)	8 (9.6%)	

Allin BSR, *et al. Arch Dis Child* 2021; 106:484–490. doi: 10.1136/archdischild-2020-320310

Yes	147 (94.2%)	75 (90.4%)	
Staged definitive procedure			0.66
No	103 (67.8%)	48 (64.9%)	
Yes	49 (32.2%)	26 (35.1%)	
First attempted approach to the pull-through			0.042
Pull-through procedure not performed prior to one year	9 (5.8%)	8 (9.6%)	
Open	57 (36.5%)	29 (34.9%)	
Laparoscopic	20 (12.8%)	21 (25.3%)	
Trans-anal	39 (25.0%)	11 (13.3%)	
Combined laparoscopic and trans-anal	31 (19.9%)	14 (16.9%)	
Rectal dissection technique			0.21
Submucosal (Soave)	81 (53.6%)	36 (48.0%)	
Peri-rectal (Swenson)	13 (8.6%)	3 (4.0%)	
Posterior (Duhamel)	57 (37.7%)	36 (48.0%)	
Weight(g) at stoma formation, median (IQR)	3622 (3040, 4200)	3550 (2700, 3800)	0.30
Weight at definitive surgery(kg), median (IQR)	5.5 (4.48, 6.64)	5.15 (4.03, 6.405)	0.32

*Percentage of those with complete data

Allin BSR, *et al. Arch Dis Child* 2021; 106:484–490. doi: 10.1136/archdischild-2020-320310
